# Supplementary figures and images for: Hemocyanin of the caenogastropod Pomacea canaliculata exhibits evolutionary differences among gastropod clades
Source: PLoS One. 2020 Jan 30;15(1):e0228325. doi: 10.1371/journal.pone.0228325 (PMC6992001; doi:10.1371/journal.pone.0228325)

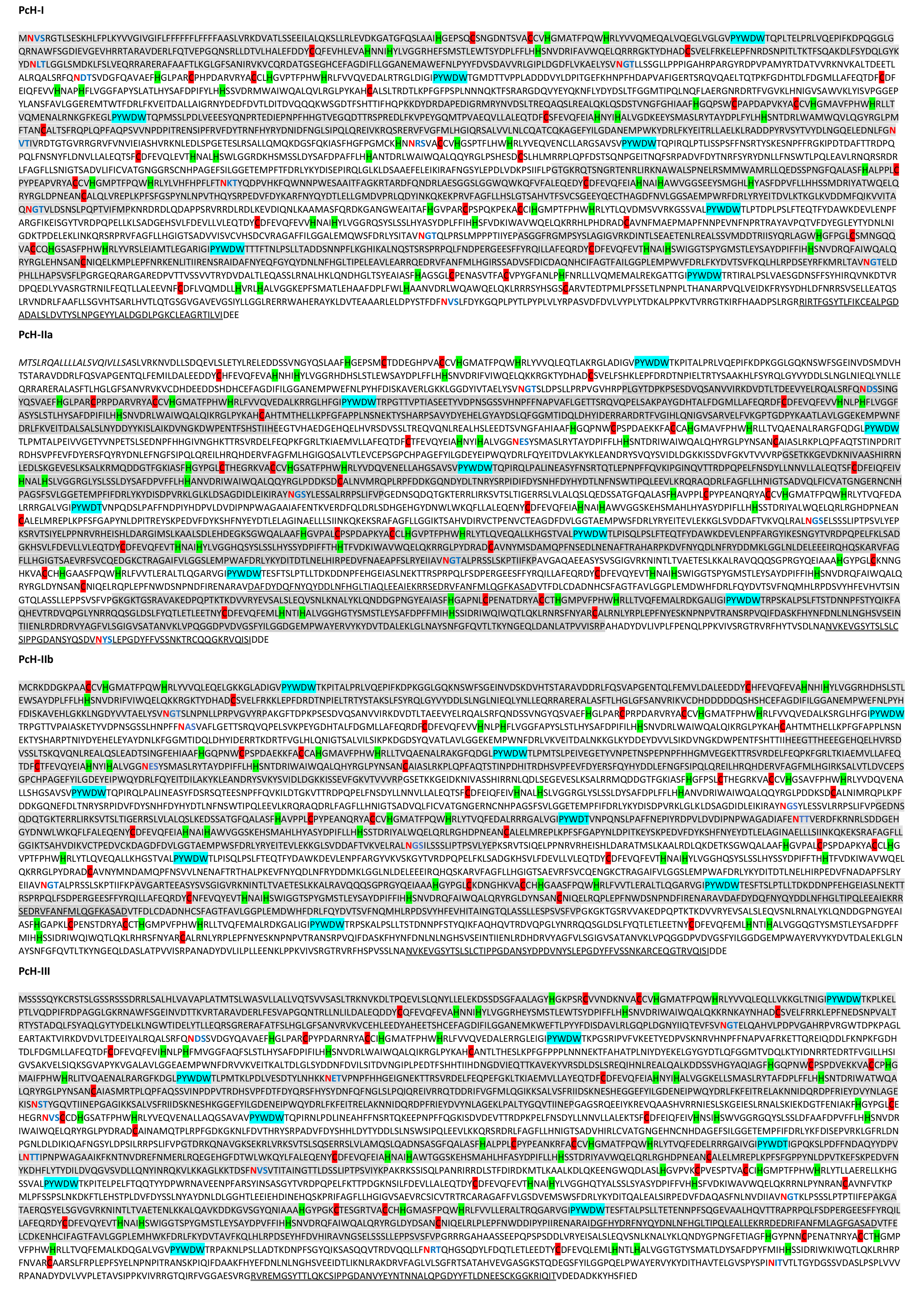

Supplement: S1 Fig — Different FUs are indicated as gray and white background sequences. Green: conserved cooper binding site Hys. Cyan: Oxygen binding motif. Red: conserved disulfide bridges. Predicted N‐glycosylated (Asn‐Xaa‐Ser/Thr) are highlighted in blue with Asn highlighted in red. Haliotisin-like domains are underlined. Cupredoxin-like domains are double underlined. (TIF) [file pone.0228325.s001.tif]

X X X PcH CCH FLH X

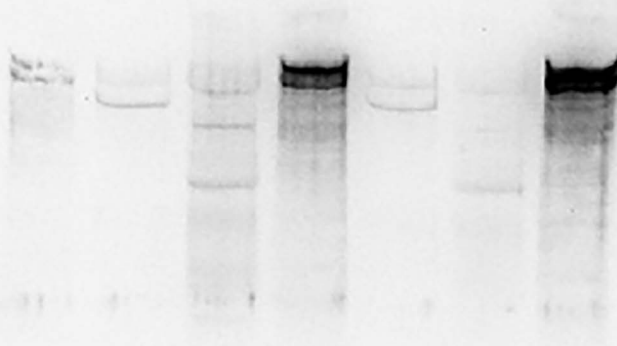

Western blot (Figure 1D)

**X X X MWM PcH CCH FLH**

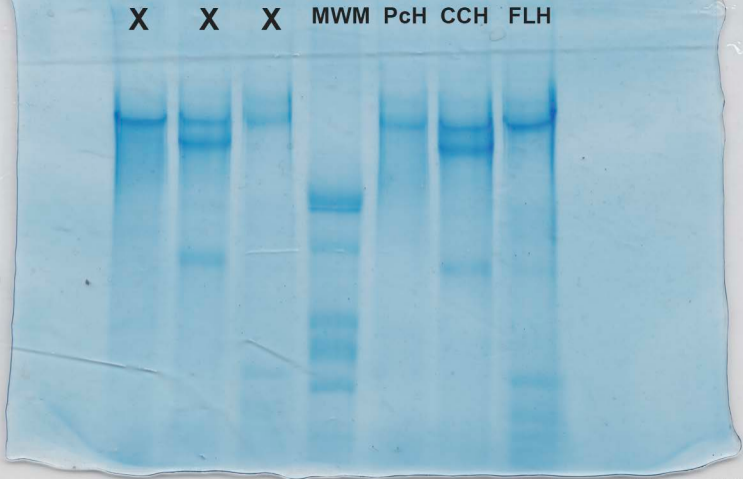

**SDS page (Figure 1C)**

Supplement: S1 Raw Images — (PDF) [file pone.0228325.s002.pdf]
