# Supplementary material for: Hemocyanin of the caenogastropod Pomacea canaliculata exhibits evolutionary differences among gastropod clades
Source: PLoS One. 2020 Jan 30;15(1):e0228325. doi: 10.1371/journal.pone.0228325 (PMC6992001; doi:10.1371/journal.pone.0228325)
Supplement: Attachment — Submitted filename: Response to Reviewers.docx [file pone.0228325.s003.docx]

La Plata, January 8^th^, 2020.

Dear Editor,

Thank you for the careful and detailed review of our manuscript **PONE-D-19-31160 “**Hemocyanin of the Caenogastropoda *Pomacea canaliculata* exhibits evolutionary differences among gastropod clades”

We apologize for the document formatting errors, these have been corrected according to the instructions for authors. Please find below the point by point response to the reviewer.

Response to reviewers.

Reviewer 1.

Minor comments,

1. Lines 133-140: The text was corrected following the reviewer’s suggestion.
2. Line134: Details on how protein concentration was calculated were added to the text as the reviewer suggested (lines 137-138 and 161).
3. Line 207 and figure 1B: TEM Negative staining images showed that PcH forms di, tri, tetra as well as multi-decameric structures. Considering 400 KDa as the subunit molecular weight, the estimated molecular weight of the smallest structure would be about 8 MDa (this estimation is now included in the text). Since the optimal separation range of the Superose-6 column we employed is 5 KDa to 5 MDa, PcH elutes in the void volume of the column. Therefore, we were not able neither to discriminate its different oligomeric structures nor to estimate their molecular weights by this technique. We have included a sentence regarding this issue in the discussion section (Lines 359-361).
4. Lines 231-242: The sentence was clarified as the reviewer suggested.
